# Supplementary material for: Patterns of Intron Gain and Loss in Fungi
Source: PLoS Biol. 2004 Nov 30;2(12):e422. doi: 10.1371/journal.pbio.0020422 (PMC532390; doi:10.1371/journal.pbio.0020422)
Supplement: Table S1 — Also available at http://genes.mit.edu/NielsenEtAl/. (4.3 MB ZIP). [file pbio.0020422.st001.zip › NielsenEtAl/html/382.html]

AN1084.1.NCU03737.1.MG08162.1.FG07182.1


```
 CLUSTAL W (1.82) Multiple Sequence Alignments - Introns Inserted


Sequence 1: MG08162.1	443 aa
Sequence 2: FG07182.1	445 aa
Sequence 3: NCU03737.1	437 aa
Sequence 4: AN1084.1	461 aa
Alignment Length: 470 aa
Number Identitical Residues: 275 aa
Alignment Score (without introns) 12454


MG08162.1 	MSATFRSLAPFARTARFAFKKT--GNPLVS-LQQQSRSPVLNLRRGYA-VYERTKPHVNI
NCU03737.1	-MSILRTIAPFMRAARAGASTN----PLMT-----MKSPMLNFNRSYASVFQRTKPHVNI
FG07182.1 	MSTAFRSVAPFLRTARQGLRANP-VNPLQSAFTKKNASGVLNLYRTYA-VFERSKPHVNI
AN1084.1  	MSGLSRTANLLFRTTKTSILRPRGVNPVQY-----ALSKDRQTVRAMATTFERTKPHVNI
          	     *:   : *::: .     . .*:         *   :  *  *:.::*:******

MG08162.1 	G1TIGHVDHGK~TTLSAAITKRQAEKGFANFLDYAAIDKAPEERKRGITISSAHIEYSTE
NCU03737.1	G~TIGHVDHGK0TTLSAAITKRQADKGLASFLDYGSIDKAPEERKRGITISTAHIEYSTD
FG07182.1 	G~TIGHVDHGK0TTLSAAITKRQADKGLANFLEYGAIDKAPEERKRGITISTAHIEYATE
AN1084.1  	G1TIGHVDHGK0TTLTAAITKHQASKGLAQFLEYGAIDKAPEERKRGITISTAHIEFSTD
          	* ********* ***:*****:**.**:*.**:*.:***************:****::*:

MG08162.1 	NRHYSHVDCPGHADYIKNMITGAANMDGAIIVVAASDGQM~PQTREHLLLARQIGIQKIV
NCU03737.1	NRHYSHVDCPGHADYIKNMITGAASMDGAIIVVAASDGQM~PQTREHLLLARQVGIQRIV
FG07182.1 	NRHYSHVDCPGHADYIKNMITGAANMDGAIIVVAASDGQM~PQTREHLLLARQVGVQRIV
AN1084.1  	NRHYAHVDCPGHADYIKNMITGAANMDGAIVVVAASDGQM2PQTREHLLLARQVGVQKIV
          	****:*******************.*****:********* ************:*:*:**

MG08162.1 	VFVNKVDALDDPEMLELVEMEMRELLNSYGFAGDDTPVIMGSALCALNDKRPEIGQQKID
NCU03737.1	VFVNKVDAIDDPEMLELVEMEMRELLSSYGFDGDETPVIMGSALCALEGKRPEIGVEKID
FG07182.1 	VFVNKVDAIDDPEMLELVEMEMRELLNTYGFEGDDTPVIMGSALMSLQNQRPEIGTQKVD
AN1084.1  	VFVNKVDAVDDPEMLELVELEMRELLNTYGFEGEETPIIFGSALCALEDRRPEIGTEQID
          	********:**********:******.:*** *::**:*:**** :*:.:***** :::*

MG08162.1 	ELLEAVDTWIPTPERDLDKPFLMSVEDVFTIGGRGTVVSGRVQRGILKRDAEIEIVGKSD
NCU03737.1	ALMQAVDDWIPTPERDLDKPFLMSVEDVFSIAGRGTVASGRVERGTLKRDQDVEIVGKGT
FG07182.1 	ELLAAVDEWIPTPERDLDKPFLMSVEDVFSIAGRGTVVSGRVERGILKRDQEIELVGKGQ
AN1084.1  	KLLEAVDTWIPTPQRDLDKPFLMSVEEVFSIPGRGTVASGRVERGLLKKDSEIEIHG-GG
          	 *: *** *****:************:**:* *****.****:** **:* ::*: * . 

MG08162.1 	EVVKTKVTDIETFKKSCEESRAGDNSGLLLRGVRREDIKRGQVIAAPGSIKAHKQFLVSL
NCU03737.1	EIIKTKVTDIETFKKSCEESRAGDNSGLLLRGIRREDIKRGMVVVAPGSVKAHTKFLVSL
FG07182.1 	EVIKTKVTDIETFKKSCEQSQAGDNSGLLIRGVRREDVRRGMVVCAPGTVKSHTQFLASL
AN1084.1  	EVQKTKVTDIETFKKSCDESRAGDNSGLLLRGTRREDVKRGMVIAAPGSIKAHKKFLVSM
          	*: **************::*:********:** ****::** *: ***::*:*.:**.*:

MG08162.1 	YVLTKEEGGRHTGFQENYMPQMYIRTA----------------------SEACSLHWPEG
NCU03737.1	YVLSKEEGGRHTGFQANYRPQMFIRSA----------------------DESVSLTFPEG
FG07182.1 	YVLTKEEGGRHTGFQEHYRPQLYLRTA----------------------DESIDLTFPEG
AN1084.1  	YVLTEAEGGRRSGFGSNYRPQAYIRTAGKSFSNNLNAIFGHHRRSNQCLDEACDLTFPDG
          	***:: ****::**  :* ** ::*:*..: :.. .:  .    :... .*: .* :*:*

MG08162.1 	TEDASSKMVMPGDNVEMTATLHSPIAVEPGLRINIREGGKTVATGLVTRILK-
NCU03737.1	TEDADSKIVQPGDNCELVATLCHPIAVEAGQRITVREGGRTVATGIITRIME-
FG07182.1 	TEDATSKMIMPGDNTEMVVTMGHPNAIEVGQRFNIREGGRTVATGLCTRIIK-
AN1084.1  	---DLSRRVMPGDNVEMILNLNRPVAAEAGQRFNIREGGRTVATGLITRVIEE
          	     *: : **** *:  .:  * * * * *:.:****:*****: **:::.
```
